# Reprogramming in vivo th17 into th17/th2 by Sirp-α dendritic cells in the lungs

**DOI:** 10.1186/1710-1492-7-S2-A27

**Published:** 2011-11-14

**Authors:** Vu Quang Van, Marianne Raymond, Keiko Wakahara, Manuel Rubio, Marika Sarfati

**Affiliations:** 1 Immunoregulation Laboratory, Centre Hospitalier de l’Université de Montréal, Research Center CRCHUM, Notre-Dame Hospital, Montréal, Québec, H2L 4M1, Canada

## Background

Dendritic cells (DCs) play a crucial role in the development of the adaptive immune response. Unbalance DC response can cause Th1, Th17 or Th2-mediated diseases. By *in vitro* manipulation, Th2 and Th17 cell lines can be reprogrammed into Th1. This highlights the notion of the plasticity of different populations of CD4 T helper cells. So far, the conversion of Th17 memory cells into Th2 cells has not been demonstrated in the tissues.

## Methods

Mice were immunized by repetitive administration of inflammatory DCs loaded with OVA protein antigen (OVA-DC), locally (intra-tracheal) or systematically (intravenous). Mice were sacrificed 24h after the last challenge and lymph nodes, serum, lungs and bronchoalveolar lavage were collected to evaluate the immune response.

## Results

We showed here, that administration of OVA-DCs generated antigen-specific CD4 T cells that produced IL-17, IL-13 and IL-4 (Th17/Th2) and expressed GATA-3 in the lungs and the lymph nodes. The immunized mice developed an IgE-independent lung inflammation that displayed resistance to treatment with corticosteroids. This inflammation was characterized by a mixed infiltration of neutrophils and eosinophils in the bronchoalveolar lavage. We demonstrated that airway inflammatory SIRP-a DCs converted *in vitro*-generated Th17 but not Th2 cell lines into Th17/Th2. Finally, passive transfer of Th17/Th2 cells was sufficient to drive airway inflammation in naïve mice.

## Conclusion

We propose that immunization with inflammatory DCs, regardless of the route of immunization, induces chronic inflammation of the airways, which is associated with a Th2/Th17 response.

